# Parasitic chyluria in a 72-year-old Sierra Leonean woman: A case report

**DOI:** 10.4102/phcfm.v11i1.2093

**Published:** 2019-10-15

**Authors:** Atilola A. Adeleke

**Affiliations:** 1 Department of Family Medicine, College of Medicine and Allied Health Sciences, University of Sierra Leone Teaching Hospital Complex, Freetown, Sierra Leone

**Keywords:** filariasis, chyluria, Sierra Leone, family medicine, West Africa

## Abstract

Although Sierra Leone lies within the worldwide filarial belt, chyluria (the passage of milky coloured urine) is a rare presenting symptom in clinical practice. This report describes a confirmed case of parasitic filariasis presenting in a 72-year-old woman. After treatment with a combination of ivermectin and albendazole, her symptoms resolved within 48 h and she was symptom free at 6 months.

## Introduction

This report describes a confirmed diagnosis of parasitic chyluria in a 72-year-old woman from rural Sierra Leone who presented with chyluria, which is the passage of milky coloured urine (chyle).^1^ Although Sierra Leone lies within the worldwide filarial belt, chyluria is an uncommon clinical presentation and a confirmed diagnosis of a parasitic cause is rare.

## Case

A.F. was a 72-year-old widow who presented with a 6-month history of passing milky colour urine. She had dysuria, but no urinary frequency, urgency, suprapubic pain or fever. She reported significant weakness, loss of appetite and weight loss. There was no swelling in any part of the body. Her past medical history was unremarkable. She resided in a village in Bombali district (about 300 km north of Freetown) all her life. She neither smoked cigarette nor drank alcohol. On account of the symptoms, she had been treated for urinary tract infection with several antibiotics to no avail necessitating the presentation at the Teaching Hospital.

### General and systemic examination revealed no significant findings

Urine dipstick revealed 2+ for protein, but negative for glucose, blood and nitrite. Urine microscopy showed epithelial cells with crystals. No organism was cultured after 48 h of incubation. Complete blood count, electrolytes, urea, creatinine, total protein and albumin were essentially normal.

The suspicion of chyluria was raised after the laboratory personnel drew the attention of the author to the separation of the urine in layers in the sample bottle. Ether test was done that clears off the urine opacity. Blood film for microfilariae showed numerous *Wuchereria bancrofti* microfilariae and fasting lipid profile showed hypocholesterolaemia. A definitive diagnosis of filarial chyluria was subsequently made.

Recommendation was made for low-fat, high-protein diet and to increase oral fluid intake to 3–4 L per day. She was commenced on oral ivermectin 9 mg stat and albendazole 400 mg daily for 14 days as diethylcarbamazine was not available. She was counselled on environmental protection towards limiting mosquito bites.

She was a widow and stayed alone. However, the District Health Management Team (DHMT) was contacted to improve on disease awareness and health education, case identification and subsequent case management at the community level (free ivermectin is made available by the government at all the primary care centres in the country).

She was seen a week thereafter with the report that the passage of milky colour urine ceased the second day after the medication. She had another dose of ivermectin 9 mg 3 weeks after the first dose and she was followed up for six consecutive months with no recurrence.

**FIGURE 1:**
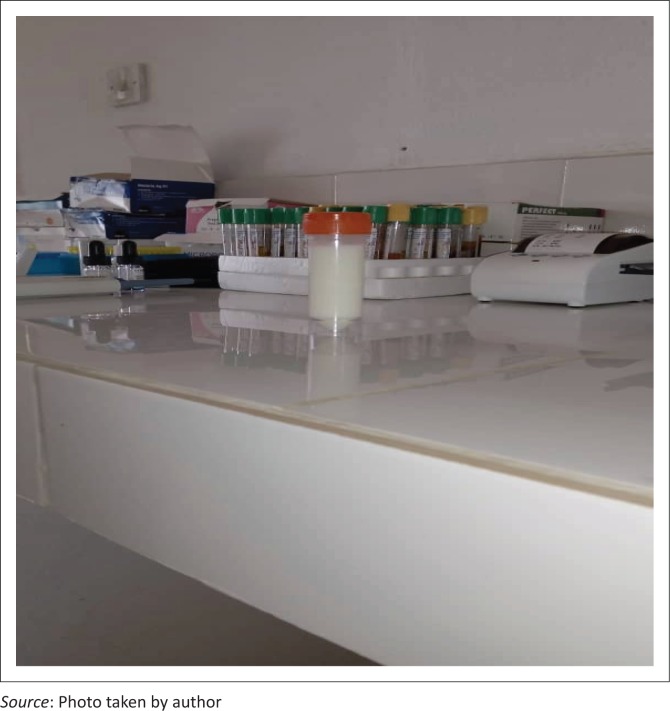
Milk colour urine sample from the index patient.

### Ethical consideration

Written informed consent was taken from the patient for reporting this case.

## Discussion

**FIGURE 2:**
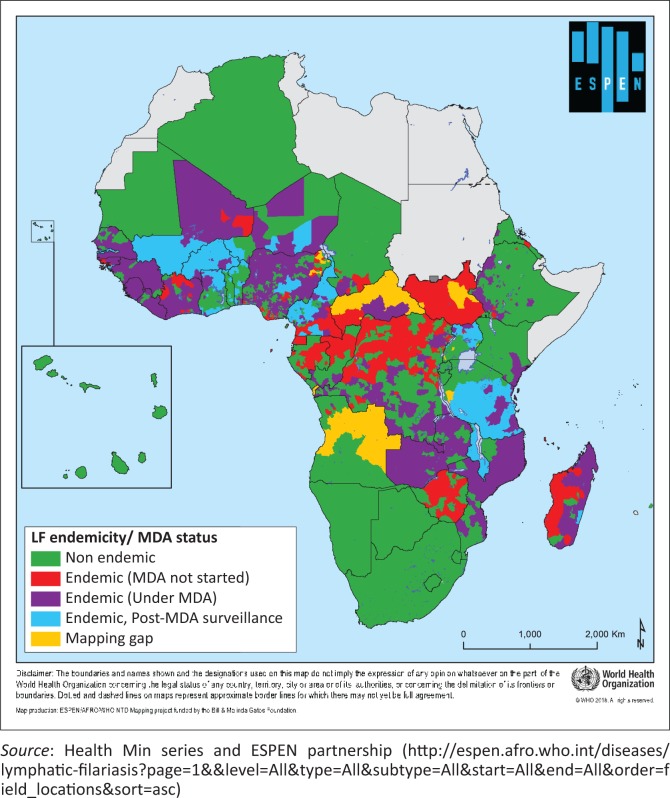
Endemicity status of Lymphatic Filariasis in WHO African region 2017.

Parasitic chyluria is endemic in south Asia and in some parts of Australia and Africa (Figure 2).

Chyluria can be classified as parasitic and non-parasitic with the former predominantly caused by adult filarial worms. *Wuchereria bancrofti* accounts for about 95% of filarial chyluria and is transmitted predominately by culex mosquitoes.^1^ Sierra Leone has a long rainy season, and humid weather favours the breeding of mosquitoes which are involved in the transmission of the microfilariae from person to person.

Other documented parasitic agents that have been reported to cause chyluria include *Echinococcus, Cysticercus cellulose, Ascaris lumbricoides, Tinea nana, Cercofrenas hominis* and malaria.^2^ The non-parasitic chyluria can be caused by congenital lymphatic aberrations, iatrogenic lympho-urinary fistulae, obstruction of the lymphatics caused by trauma (mainly after partial nephrectomy), abscess, neoplasms (acute myeloid leukaemia and testicular malignancy), diabetes, pregnancy and infections like tuberculosis, leprosy and mycosis.^2^

In converse to the male preponderance in most studies, the index case was a female^2,3,4,5^ and her age of 72 years is at variance with the age bracket of the majority of affected patients, which falls between 15 and 30 years.^2,4,6^

The pathogenesis of parasitic chyluria is obstruction of the major intestinal lymphatic and thoracic duct by the mature filarial worms. The subsequent build up of lymph behind the gut and pelvic lymphatic obstruction, as well as the local inflammatory reaction to the filarial worms, leads to the leaking of lymph to the urinary tract through an abnormal connection between the lymphatic system and the urinary tract.^3^ Another theory postulates that toxins released by the dying filarial worms lead to the wall of the lymphatic vessels, which ultimately lead to impairment of the mechanism of the lymphatic valve and subsequent rupture into the urinary tract.^2,3,4^

The principal clinical features of passage of milky colour urine, proteinuria and hypocholesterolaemia seen in the index case were consistent with the findings reported in most studies.^1,2,3,4^ However, the expected hypoproteinaemia was not seen in the patient, which might indicate a milder form of the condition. Symptoms indicating more severe disease might include flank pain, difficulty in bladder emptying from bladder clots in severe cases, frequency, haematuria, backpain, malnutrition and hypercoagulability.^1,2^

To confirm the presence of chyle in the urine, a urine sample is allowed to settle down in a test tube and three layers will be observed: fatty top layer, fibrinous middle layer and debris in the bottom layer.^4^ Under microscope with dark ground illumination, chylomicrons can be visualised; chylomicrons also stained with Sudan III that was, however, not available for use in the index case.^5^ Fat solvent-ether, as used in the index case, clears off the top layer and is appropriate in resource-poor settings.^6^ Quantitatively, urinary triglyceride measurement can be done using biochemical analyser or photoelectric calorimeter.^6^

Methods of detecting the abnormal connection between the lymphatic system and the urinary tract in higher resource settings include lympho-urinary fistulae cystourethroscopy, retrograde pyelography, lymphography, lymphoscintigraphy and computer tomography scan, but those were not available as at the time the patient was seen.^4,7^

A low-fat diet is recommended to reduce chyle absorption from the intestine, thus reducing intra-lymphatic pressure. The drug of choice in filarial chyluria is diethylcarbamazine given at 6 mg/kg/day for 21 days.^1^ Alternatively, a single dose of ivermectin between 6 mg and 12 mg and repeated after 3 weeks can be used in situation where side effects of diethlycarbamazine are reported. Combination of diethylcarbamazine and/or ivermectin with albendazole 400 mg/day for 14 days has been showed to improve the clearance of the microfilariae from the circulation as seen in the index case.^1^

In contrast with reports of cases requiring repeated treatment,^2^ the disappearance of symptoms in the index case was noticed on the second day after commencing the medications, which is interesting but unexplained. Against high recurrence within the first 6 months of commencing antifilarial, the index case was followed up for 6 months and there was no recurrence of the symptoms.

Diethylcarbamazine can be repeated if there is poor response to the initial treatment after 6–8 weeks.^2^ Doxycycline has been used with success in situation where response to diethylcarbamazine has been poor.^8^ However, invasive modalities is indicated if there is no clinical improvement after two to three courses.^2^

Tanaka et al. reported a successful treatment of massive proteinuria and severe chyluria with Ezetimibe, a cholesterol-lowering drug, which was believed to work by reducing chyle absorption from the gut and preventing rupture of the lymphatic vessels into the urinary tract.^9^

Sclerotherapy which involves instilling 1% silver nitrate or povidone iodine (2 mL with 8 mL distilled water) has been shown to be effective.^10^ Surgical methods are indicated in patient with refractory severe chyluria and severe proteinuria, hypo-proteinuria, progressive weight loss and immunodeficient state from hypogammaglobulinemia.^9^

Potential complications of parasitic chyluria include severe malnutrition from hypoproteinaemia and fat-soluble vitamin deficiency, immunosuppression, depression especially from chronic chyluria, recurrent renal colics and so on.^9,10^

Differential diagnoses of parasitic chyluria include urinary tract infection, particularly pseudomonas pyuria, severe nephrotic syndrome and other causes of non-parasitic chyluria that have earlier mentioned.^2,11^

## Conclusion

Chyluria is a relatively rare manifestation of filariasis in Sierra Leone that may be because of under-reporting, misdiagnosis or reluctant health-seeking attitude of the affected population. It is, however, pertinent for healthcare personnel within the region to consider it as a possible differential in any patient with milky urine and to investigate appropriately. In this case, the combination of Ivermectin and Albendazole resolved symptoms within 2 days and this may be something that requires further research, although it is possible that the absence of hypoproteinaemia indicates that her case was not severe.

Beyond case management, frontline physicians should equally see every case as an opportunity to reach out to the family and the community at large.
